# Hybrid ST-ResNet and LSTM approach for precise crime hotspot prediction

**DOI:** 10.1038/s41598-025-24559-7

**Published:** 2025-11-19

**Authors:** Nasim Shahmoradi, Ali Asghar Alesheikh, Ali Jafari, Aynaz Lotfata

**Affiliations:** 1 Department of Geospatial Information Systems, Faculty of Geodesy and Geomatics Engineering, K. N. Toosi University of Technology, Tehran, Iran; 2 Geospatial Big Data Computations and Internet of Things (IoT) Lab, K. N. Toosi University of Technology, Tehran, Iran; 3 Department of Pathology, Microbiology, and Immunology, School of Veterinary Medicine, University of California, Davis, Davis, USA

**Keywords:** Real-time crime prediction, Hotspot mapping, Spatio-temporal analysis, ST-ResNet, LSTM networks, Environmental social sciences, Engineering

## Abstract

Crime is a critical social issue that threatens public safety, and accurately predicting and reducing crime in hotspot areas is a key objective. Crime patterns are inherently complex, with substantial spatial and temporal variability, which makes accurate prediction challenging. This study addresses these intricacies by leveraging advanced deep learning models, including Convolutional Neural Networks (CNNs) and Recurrent Neural Networks (RNNs), to enhance predictive accuracy in crime hotspots. Specifically, we integrate ST-ResNet for spatio-temporal analysis with Long Short-Term Memory (LSTM) networks to model temporal dependencies, overcoming ST-ResNet’s limitation in capturing sequential patterns in crime data. To further improve predictive accuracy, we introduce a feature: the daily Euclidean distance of each crime to the nearest park as a secondary input channel for ST-ResNet, supplemented by historical crime data. Additionally, weather conditions and temporal variables are incorporated into the model’s fully connected layer as additional inputs. Our method achieves a mean hit rate of over 88% at finer spatial resolutions (500 m), outperforming existing models, which tend to perform better only at coarser resolutions (1000 m). By capturing finer spatial details, our approach proves more effective in identifying theft hotspots across Chicago, offering valuable insights for developing targeted crime prevention strategies.

**Supplementary Information:**

The online version contains supplementary material available at 10.1038/s41598-025-24559-7.

## Introduction

Crime is one of the most critical threats to urban security and residents’ quality of life. In the United States, the Federal Bureau of Investigation (FBI) reported more than 1.2 million violent crimes in 2017, with theft-related losses amounting to billions of dollars^1^. This substantial social and economic burden underscores the urgent need for accurate crime prediction. Accurately identifying hotspots and predicting the time and location of criminal incidents allows law enforcement to allocate resources efficiently, intervene before crimes occur, and strengthen public safety.

Classical criminology theories emphasize the spatio-temporal nature of crime patterns^2,3^. However, early crime prediction studies often examined either temporal^4^, or spatial factors^5,6^. Given that crime varies across time and space, integrating both dimensions is crucial for accurate forecasting^7^. In this context, deep learning is particularly effective, capturing complex spatio-temporal patterns through robust feature representation^8^. In particular, Convolutional Neural Networks (CNNs) are widely employed to extract spatial features, while Recurrent Neural Networks (RNNs) model temporal sequences, together offering complementary strengths for spatio-temporal prediction^8,9^.

Achieving precise crime prediction remains difficult due to spatial complexities, where crimes are influenced not only by local clustering in neighboring regions but also by broader, distant dependencies^10^. Additionally, many prior studies have focused on large administrative units or coarse grid systems, capturing broad spatial patterns but failing to reflect finer local variations. For example, Wang et al.^11^ used a fixed 17.8 km grid, which lacks resolution at the neighborhood level. In contrast, Zhang et al.^12^ showed through a multi-scale study that medium grids (around 2.4 km) outperform smaller ones (1.2 km). These findings suggest that although finer spatial resolutions are theoretically more suitable for detailed analysis, they often suffer from data sparsity, particularly in low-crime areas where events are highly scattered^13^.

Besides, in the temporal domain, crimes often exhibit recurring daily and weekly patterns^14^, which need to be modeled alongside sequential dependencies. Although models such as the Spatio-Temporal Residual Network (ST-ResNet) and Spatio-Temporal Graph Convolutional Network (ST-GCN) have been applied to spatio-temporal prediction^11,15,16^, their treatment of time as parallel channels limits their ability to capture sequential order, thereby reducing predictive accuracy. Moreover, most studies have emphasized yearly, weekly, or monthly scales, while attempts at daily or hourly prediction often suffer from high error rates, measured by Root Mean Square Error (RMSE) and Mean Absolute Percentage Error (MAPE)^12,14^. Yet, daily-level prediction remains crucial for practical applications such as patrol scheduling and security inspections.

In addition, external factors can strongly affect crime rates. Previous studies have mostly relied on static temporal features such as weather conditions^17^ or calendar indicators (e.g., weekdays, weekends, holidays), which do not change across locations and therefore fail to capture the dynamic nature of urban environments. Therefore, there is a need for models that integrate truly dynamic spatio-temporal features—beyond static weather or calendar variables—to better reflect the evolving urban environment and its influence on crime.

In response to these gaps, this study proposes a multi-stage deep learning framework that integrates both static and dynamic spatio-temporal features. Specifically, the Diurnal Periodic Integral Mapping (DPIM) method is used to reduce data sparsity at finer spatial scales. By aggregating crime events, DPIM ensures more uniform data distribution and mitigates the sparse data problem in small grids, thereby enhancing the robustness of the model and improving the reliability of predictions across diverse urban environments. The main contributions of this research are as follows:

1. First use of park proximity as a dynamic feature in crime prediction, directly capturing the influence of urban green spaces on crime distribution.
2. Integration of static and dynamic auxiliary variables to enrich the spatio-temporal context.
3. Enhancement of the ST-ResNet architecture with Long Short-Term Memory (LSTM) units and attention mechanisms, enabling the model to better capture sequential temporal dependencies and remain robust under sparse and irregular crime data.
4. First systematic evaluation across three spatial scales (2000 m, 1000 m, and 500 m), showing that, unlike prior state-of-the-art (SOTA) baselines which peak at 1000 m, the proposed model uniquely sustains the highest hotspot prediction accuracy at 500 m resolution—the scale most relevant for operational policing.

## Related works

Crime prediction methods have evolved alongside technological progress. Early analyses relied on Geographic Information Systems (GIS), which provided valuable spatial insights but lacked predictive capability. To address this gap, traditional statistical models such as bayesian regression and Autoregressive Integrated Moving Average (ARIMA) were applied^18,19^. With advances in computational power, machine learning algorithms including Support Vector Machines (SVM), k-Nearest Neighbors (KNN), decision trees, and ensemble approaches such as Gradient Boosting and XGBoost demonstrated improved accuracy in hotspot detection and crime trend analysis^20–22^. Nevertheless, these models remain limited due to their dependence on manual parameter tuning and their reduced robustness when facing sparse or noisy data^23,24^.

Deep learning, as a branch of machine learning, has created a major breakthrough in crime prediction, as it can capture complex spatiotemporal dependencies, surpass traditional methods, and reduce the need for manual intervention^25^. Numerous studies have employed deep learning networks for crime prediction across different spatial scales^11,12,14^. Some approaches focused on single-scale resolutions. For example, Wang et al.^11^ applied the ST-ResNet architecture without hierarchical decomposition, modeling all crimes in Los Angeles on a fixed grid with a resolution of 17.8 km. In this framework, static spatial variables such as weather and calendar holidays were added to the model through an external Fully Connected layer, but the lack of a temporal module prevented proper modeling of time sequences. Subsequently, Zhang et al.^12^ evaluated the same architecture at resolutions of 9.6, 4.8, 2.4, and 1.2 km, demonstrating that the 2.4 km resolution yielded the best accuracy with RMSE = 7.81. Similarly, Zubair et al.^26^ used a GCN to predict crime locations in Chicago with grid cells of 2.2 × 2.2 km, but due to the absence of a temporal network, the model was restricted to the spatial dimension. Likewise, Caffaro et al.^27^ trained a transformer model on monthly Boston crime data using a 1 km resolution grid, without incorporating external data or dynamic features. In contrast, Fan et al.^28^ combined Informer + ST-GCN to predict daily crimes in Chicago, dividing the city into 22 police districts and incorporating static variables such as weather and holidays along with historical crime data.

In contrast to these single-scale studies, some research adopted multi-scale approaches. For example, Tekin & Kozat^29^ developed a Graph Convolutional Gated Recurrent Unit (Graph-ConvGRU) combined with a Multilayer Perceptron (MLP) at a base resolution of 1 km, and to handle sparse data, subdivided cells with recorded crimes into finer scales while keeping crime-free cells unchanged. In this model, calendar features such as holidays and weekends were also included as inputs. However, the use of multilayer Graph-ConvGRU greatly increased computational and memory costs, making performance dependent on threshold choices and minimum cell size. Jing et al.^13^ also introduced the Spatio-Temporal Hierarchical Gating Network (ST-HGNet) for predicting assault crimes in Chicago. This model was tested at three scales (500, 1000, and 2000 m) and achieved the best performance at 1000 m resolution with an average accuracy of 84%. This study only used crime data without external variables.

The literature review shows that the trajectory of research, from simple statistical models to multi-scale deep learning, has consistently aimed to improve accuracy while capturing both spatial and temporal dimensions. However, most studies have either remained limited to coarse spatial and temporal scales or relied solely on static variables such as weather and calendar indicators, failing to capture the dynamic nature of urban environments. More recent attempts using advanced models like ST-ResNet, ST-GCN, or Transformer-based approaches have improved spatio-temporal modeling but often lacked sequential temporal modules or excluded dynamic external features, leading to reduced accuracy at finer resolutions (Table 1). This highlights the need for frameworks that simultaneously integrate sequential temporal modeling (e.g., via LSTM), attention mechanisms for feature reweighting, and dynamic spatio-temporal features—such as daily park proximity—into enhanced architectures like ST-ResNet, in order to deliver accurate yet computationally efficient predictions at local and daily scales.

**Table 1:** Summary of deep learning approaches for crime prediction with input data, resolution, and limitations.

Authors	Technique	Study area	Input data	Resolution (spatial / temporal)	Attention	Limitation
Wang et al.^11^	ST-ResNet	Los Angeles	Crime counts + weather + holidays	17.8 km/ hourly	✗	Coarse spatial scale; Lack sequential temporal modeling; No dynamic spatio-temporal features
Zhang et al.^12^	ST-ResNet	Suzhou, China	Crime counts + temperature	9.6, 4.8, 2.4, 1.2 km / hourly	✗	Coarse spatial scale; Lack sequential temporal modeling; No dynamic spatio-temporal features
Tekin & Kozat^29^	Graph-ConvGRU + MLP	Chicago	Crime counts + calendar (weekends, holidays)	1 km/ daily	✗	Coarse spatial scale; No dynamic spatio-temporal features
Jing et al.^13^	Spatio-temporal Hierarchical Gating Network (ST-HGNet)	Chicago	Historical assault crimes	2000 –500 m/ daily	✓	limited accuracy at finer scale (500 m); No dynamic spatio-temporal features
Caffaro et al.^27^	Transformer	Boston	Crime counts	1 km/ monthly	✓	Coarse spatio-temporal scale, No dynamic spatio-temporal features
Fan et al.^28^	ST_GCN + Informer	Chicago	Crime counts + weather + holidays	Police district/ daily	✓	Coarse spatio-temporal scale; No dynamic spatio-temporal features
Zubair et al.^26^	GCN	Chicago	Crime counts	Nodes correspond to 2.2 km × 2.2 km grid cells/ ---	✗	Lack temporal prediction

## Study area

**Fig. 1:**
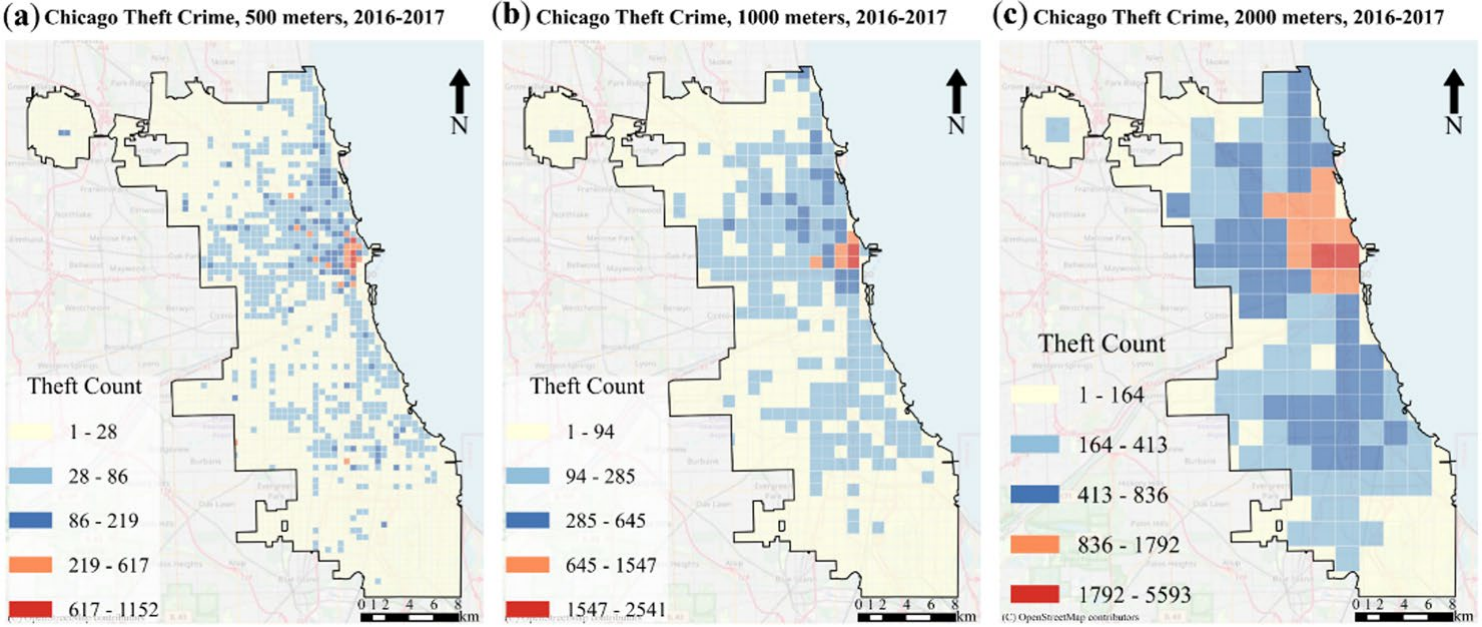
Study area at different spatial resolutions: (**a**) 500, (**b**) 1000, and (**c**) 2000 m using Google Street View.

Our study focuses on crime prediction modeling in Chicago, Illinois, (41.88° N, 87.63° W), the third-most populous U.S. city with over 2.7 million residents^30^. Known for its diverse urban landscape and high crime rates—particularly theft, one of its most frequently reported offenses^31^. These characteristics make it an ideal location for testing and refining crime prediction models. Figure 1a-c illustrates the study area and the spatial distribution of theft crimes (2016 to 2017) in Chicago.

This study adopts a city-wide approach to predicting crime for several reasons: data is more comprehensive and accessible at this scale; specific crime patterns extend beyond neighborhood boundaries and are best analyzed city-wide; many crime patterns transcend neighborhood boundaries; and city-wide models support more effective decision-making in law enforcement operations, including patrol planning and resource allocation.

## Materials and methods

### Data

#### Crime data as an outcome variable

The data for training our models and conducting experimental evaluations are sourced from the Chicago Open Data Portal^32^. This portal, developed and managed by the Chicago government, offers comprehensive datasets, maps, and graphs about the city, with a publicly available download service.

Daily theft crime data encompassing 242,770 records over a span of 1,096 days (from January 1, 2014, to December 31, 2016) was utilized as the dataset source. The analysis focused on event distribution across 2000, 1000, and 500-meter grid resolutions within the specified geographical bounds of [41.64°, 42.02°] latitude and [-87.93°, -87.52°] longitude.

The 2000-meter and 1000-meter grid sizes were selected based on prior research^33,34^ demonstrating their effectiveness in analyzing theft crimes^35^. Our study introduced the 500-meter grid resolution to analyze theft crimes in Chicago. While this finer resolution has been applied in the analysis of other crimes, such as assaults^13^, its application to theft crimes in Chicago represents a novel approach to our research. This 500-meter grid allowed us to capture more detailed spatial patterns, which were crucial for generating the matrix datasets needed for deep learning-based predictions. The results of this spatial partitioning at various resolutions are summarized in Table 2.

**Table 2:** The results of partitioning.

Spatial Resolution	Row	Column
2000 m	21	18
1000 m	42	35
500 m	84	70

#### Covariates

In addition to the crime data, we incorporated several covariates to enrich our analysis.

#### Weather

For over 150 years, researchers have studied the relationship between weather and crime^36,37^. Although scholarly interest in this area has a long history, the past decade has seen a significant rise in the number and variety of empirical studies exploring the social implications of weather, including its impact on crime^38^. In our study, we collected daily weather data for the entire city of Chicago^39^, in tabular format, incorporating variables such as temperature (measured in degrees Celsius), wind speed (measured in kilometers per hour), and nine specific weather conditions (Snow-Overcast, Snow-Partially Cloudy, Partially Cloudy, Snow-Rain-Partially Cloudy, Snow-Rain-Overcast, Rain-Overcast, Clear, Rain-Partially Cloudy, and Overcast) that occurred between 2014 and 2016. This comprehensive inclusion of weather factors, such as temperature^40^ and wind speed^41^, enables us to explore potential correlations between varying weather conditions and the frequency or severity of theft occurrences.

#### Weekdays and weekends

Each theft incident was assigned a specific day to capture temporal variations in crime patterns. We categorized the incidents into weekdays, weekends, and holidays, with holiday dates determined using the official Chicago calendar.

#### Parks

In this study, we also utilized the parks dataset extracted from the Chicago Data Portal^42^. This dataset includes the geographical locations of parks across the city of Chicago. To enhance the predictive accuracy of our model, we calculated the daily Euclidean distance from each theft incident to the nearest park. This distance metric was incorporated as an additional input in the ST-ResNet model, alongside crime data, to explore its potential influence on theft occurrences.

### Methods

This section will outline the mathematical framework for crime prediction and explain the developed model.

#### Problem definition

This study aims to predict crime occurrences across various locations within a specified timeframe, mainly focusing on hot spots. To achieve this, the study area is divided into *I*$~ \times$ *J* sections based on geographical coordinates. Each section is a square with a side length of *W*. The geographical location is precisely specified according to the grid division, denoted by row number and column number as *L*_*i, j*_ where *{i = 0*,* 1*,* 2*,* …*,* I* and *j = 0*,* 1*,* 2*,* …*,* J}*. Let $x_{{{\text{i}},\ {j}}}^{t} \in {\mathbb{N}}$ denote the number of crimes occurring in each section, where the time interval *t* ranges from a few hours to a day or longer. This framework effectively transforms the data into a spatio-temporal format, capturing both the spatial distribution of crimes and their temporal variations. Consequently, the crime distribution over this interval for the entire area can be expressed as a matrix: ${X^t}$ *= [*$x_{{{\text{i}},\ {j}}}^{t}$*].* Assuming that the total number of time intervals is *T*, the set of crime distribution matrices is: *X = {*${X^t}$*}; t = 0*,* 1*,* …*,* T*. The objective is to predict the crime distribution for the next time interval *(*${X^{T+1}}$*).*

#### Model overview

Crime data is modeled using an LSTM network^43^, a type of RNN that efficiently captures long-term temporal dependencies. LSTMs maintain relevant historical information by selectively updating memory cells, mitigating issues like vanishing gradients. While effective for temporal analysis, this approach does not inherently incorporate spatial dependencies.

To address the spatial dependencies, we turn to CNNs^44^, which excel at capturing spatial relationships within data. While simple CNNs effectively model nearby spatial dependencies, modeling distant dependencies typically requires a deep network with many consecutive convolutional layers^15^. However, as the network depth increases, training accuracy can start to decrease due to the vanishing gradient problem^45^. To overcome this challenge, our model incorporates Deep Residual Learning^46^, a proven effective technique in training deep neural networks. Deep Residual Networks (DRNs), composed of many Residual Units, have established deep architectures that exhibit high accuracy and robust convergence in various applications, including classification, object detection, segmentation, and localization^47^. DRNs effectively identify nearby and distant spatial dependencies as the network depth increases. However, DRNs are primarily suited for capturing spatial dependencies.

Given the strengths and limitations of LSTM and DRNs, our research on crime prediction—particularly at finer spatial resolutions on a daily basis—utilizes a framework that integrates ST-ResNet and LSTM. ST-ResNet, introduced by Zhang and his colleagues^16^, is effective at capturing spatial patterns through multiple ResNets, each processing spatial data from different time steps as separate branches. This setup enables the identification of both long-term and short-term spatial dependencies. However, ST-ResNet al.one is limited in modeling sequential temporal dependencies because it concatenates data fragments into a single tensor, weakening the temporal relationships.

To address this, we propose a hybrid approach that combines ST-ResNet with LSTM. While ST-ResNet excels in capturing spatial relationships, LSTM enhances the model’s ability to learn and represent temporal patterns, improving the accuracy of crime predictions across various locations and times. Our focus is on daily crime prediction, as short-term forecasting is more relevant for most police departments^12^. Crimes like burglary and theft are often transient and require rapid responses to prevent and address effectively^48^. The current research gap in short-term prediction models limits proactive crime prevention efforts, such as optimizing patrol routes and deploying officers to specific areas. Additionally, our models incorporate variables such as weather conditions and proximity to parks to further enhance prediction accuracy. The following sections will detail the architecture and components of the proposed network.

#### Framework overview

**Fig. 2:**
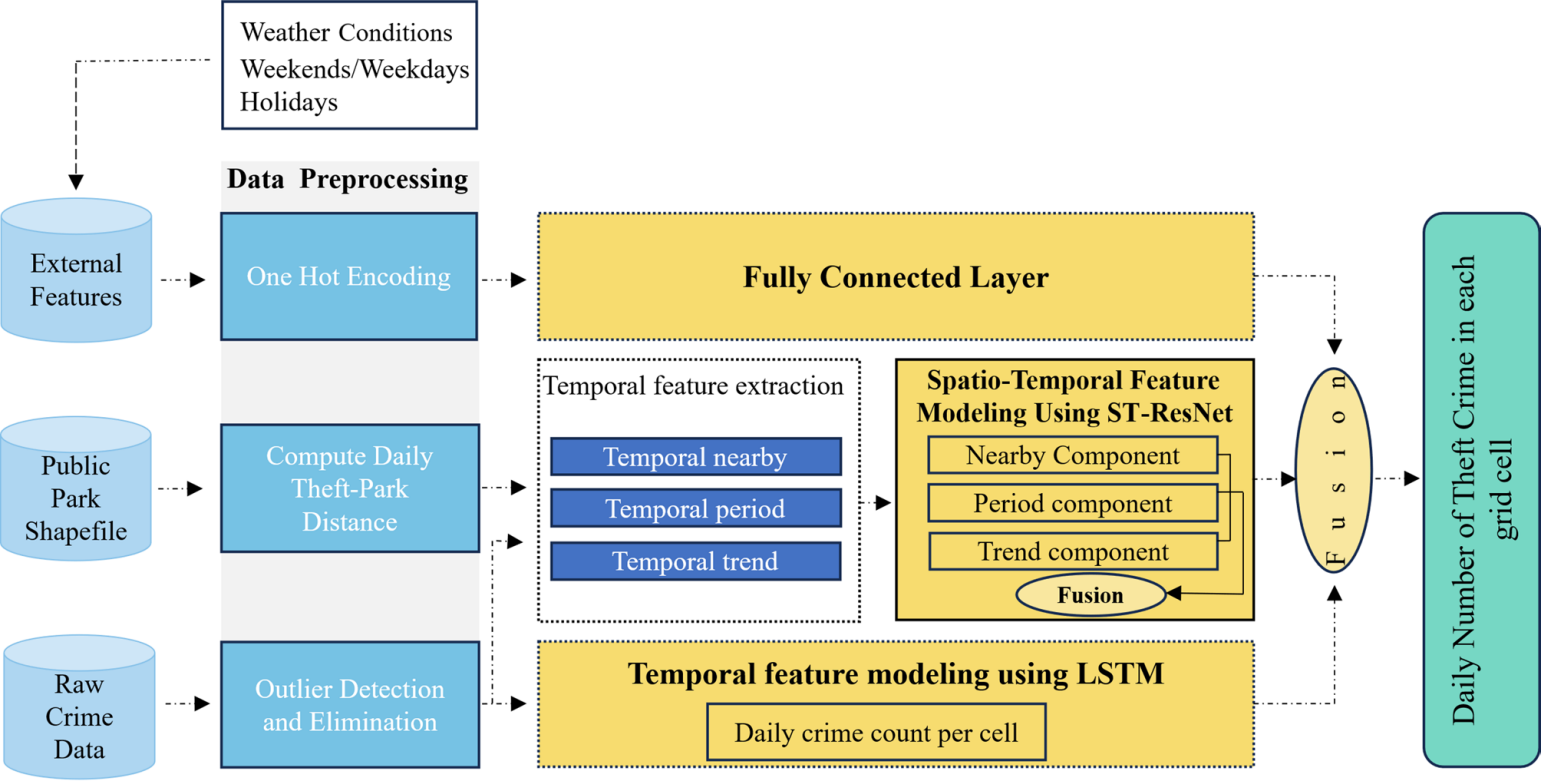
Workflow framework.

As detailed in the introduction section, Crime is a multifaceted phenomenon influenced by various economic, political, and periodic patterns. These complexities necessitate a comprehensive model that can effectively capture these dependencies. The proposed model addresses this need by incorporating components to model spatial, temporal, and external dependencies. As depicted in Fig. 2, the model inputs are raw historical crime records, public park locations, and external features, and it outputs the predicted daily number of theft crimes in each grid cell. The framework consists of four main components: data preprocessing, spatio-temporal feature extraction (ST-ResNet), temporal feature extraction (LSTM), and a fully connected layer for integrating external features.

#### Data preprocessing

The crime data reported by relevant authorities includes information on the type, time, and location of crimes. In the initial step of the data preparation, outlier data points situated outside the designated study area were excluded while maintaining temporal continuity. Subsequently, considering the desired time interval for prediction and the zoning of the geographical study area, the data can be converted into image-like matrices as, ${X^t}$ = [$x_{{{\text{i}},j}}^{t}$]. As the selected time interval becomes smaller, the crime data matrix becomes sparser and more irregular, complicating the task for deep learning models. To address this issue, we utilize the DPIM method, as outlined in Eq. (1), to adjust the data accordingly:

1 $${Y^t}{\text{}}={\text{}}\mathop \smallint \limits_{{{\text{i}}={\text{t}} - {\text{k}}\Delta {\text{t}}}}^{{\text{t}}} {{\text{X}}^{\text{i}}}$$

In Eq. (1), ${X^{\text{i}}},{\text{}}$ represents the crime data matrix for the *i*-th time interval, and ${Y^t}$denotes the cumulative crime data matrix from the time interval t - k$\:\varDelta\:t$. To capture temporal dependencies, the crime count for each subsequent day was updated by adding the previous day’s count. This sequential summation was applied to the entire dataset, comprehensively representing temporal dynamics. By integrating these strategies, we balanced addressing sparsity concerns in the early stages and capturing the dynamic nature of crime occurrences, providing a solid foundation for subsequent analyses and modeling efforts. We also applied Min-Max normalization to keep the data consistent.

In this study, to model the dynamic relationship between crimes and parks, we have developed a novel method for generating park data and using it alongside crime data as a second channel in our deep learning network. To our knowledge, this method has not been previously utilized in any other study. Each crime is associated with a distance to the nearest park, calculated based on the crime’s location and the locations of parks within the study area. There is a corresponding distance to the nearest park for each crime occurring in the t-th time interval in the region L_i_,_j_. If d_i_,_j_ represents these distances, the distance matrix for the entire area in the t-th interval can be defined as ${D^t}$= [$d_{{{\text{i}},\ {j}}}^{t}$]. According to this definition, for each crime matrix,$~{Y^t}$formed for the t-th time interval, the corresponding distance matrix ${D^t}$ is also formed. In forming the ${D^t}$ matrices, both the location of the parks and the potential crime density around them in different time intervals are considered. This modeling approach can effectively capture the dynamic relationship between crimes and parks.

The crime data was refined and structured through these preprocessing steps for robust hotspot predicting in Chicago. The resulting aggregated crime counts, organized by spatial grids and temporal sequences, provide a foundation for subsequent predictive modeling and analysis.

#### Spatio_Temporal feature module

**Fig. 3:**
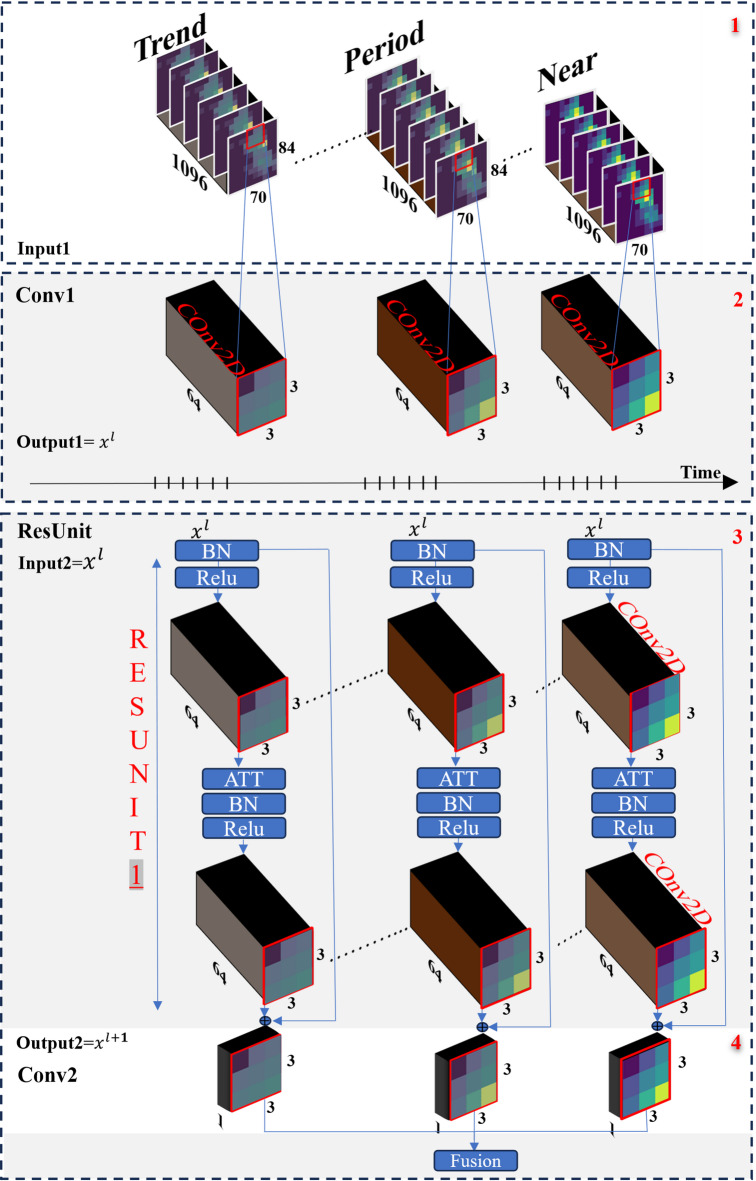
Proposed ST-ResNet with Attention-enhanced Residual Units (ResUnits include batch normalization (BN), ReLU, Conv, and attention (ATT).

The spatio-temporal feature extraction module is based on ST-ResNet, which integrates CNNs with residual learning to capture both local and distant spatial dependencies across the city grid. As shown in Fig. 3, the module consists of three main components—Near, Period, and Trend—that encode short-term, periodic, and long-term crime patterns. For each component, crime data and park proximity information are combined to form a two-channel input matrix.

Each component follows a similar residual architecture, beginning and ending with convolutional layers. At the core of the design are multiple ResUnits. In proposed framework, we extend each ResUnit with an Attention block (ATT) positioned after the convolutional layers. The attention mechanism adaptively emphasizes salient spatio-temporal features while suppressing less informative signals, thereby improving the model’s robustness in detecting crime hotspots, particularly under sparse and irregular patterns. The updated ResUnit structure, referred to as an Attention-Enhanced ResUnit, is illustrated in Fig. 3.

According to the data preprocessing section, daily crime counts and their distances to parks are represented as two-dimensional matrices ${Y^t}$​ and ${D^t}$ based on the city grid. The Closeness branch uses recent data near the prediction time, specifically crimes and their distances to the nearest park, represented as : [${Y^{t{\text{}} - {\text{}}lc.c}}$, ${Y^{t - \left( {lc - 1} \right).c}}$, …, ${Y^{t{\text{}} - {\text{}}1}}$] ; [${D^{t{\text{}} - {\text{}}lc.c}}$, ${D^{t - \left( {lc - 1} \right).c}}$, …, ${D^{t{\text{}} - {\text{}}c}}$]. These data points concatenate along with the first axis (i.e., time interval) into a single tensor.

Similarly, the Period and Trend branches are structured like the Closeness branch, but with periodic and trend intervals. For the Period branch, considering *lp* as the periodic interval, the inputs are: [${Y^{t~ - ~lp.p}}$, ${Y^{t - \left( {lp - 1} \right).p}}$, *…*, ${Y^{t~ - ~p}}$] ; [${D^{t~ - ~lp.p}}$, ${D^{t - \left( {lp - 1} \right).p}}$, *…*, ${D^{t~ - ~p}}$]. For the Trend branch, considering *lq* as the trend interval, the inputs are: *[*${Y^{t~ - ~lq.q}}$, ${Y^{t - \left( {lq - 1} \right).q}}$, *…*, ${Y^{t~ - ~q}}$*]* ; *[*${D^{t~ - ~lq.q}}$, ${D^{t - \left( {lq - 1} \right).q}}$, *…*, ${D^{t~ - ~q}}$*].*

The values *lc*, *lp*, and *lq* represent the number of time intervals for the Near, Period, and Trend fragments, respectively, while *c*, *p*, and *q* denote the corresponding spans. According to^14^, these numbers are typically set to 3 with *c*, *p*, and *q* corresponding to a day, a week, and a year, respectively.

The outputs of the three networks are denoted as *O*_*n*_, *O*_*p*,_ and O_t_ ​. Given the varying temporal impacts of each network, a weighted sum of these outputs is used to determine the final result, as expressed in Eq. (2):

2 $${O_{Res}}={\text{ }}{W_n}*{\text{ }}{O_n}+{\text{ }}{W_p}*{\text{ }}{O_p}+{\text{ }}{W_t}*{\text{ }}{O_t}$$

In Eq. (2), the symbol “*” denotes element-wise multiplication. The parameters *W*_*n*_ ، *W*_*p*_ ، *W*_*t*_ ​ are learnable weights that are adjusted during network training.

#### Temporal feature module

Crime exhibits spatial and temporal characteristics, requiring temporal analysis for accurate prediction. This model uses LSTM networks to extract temporal features.

The network’s input consists of crime data over *n* days. Representing the daily crime matrices for the *n* days preceding the prediction day as: [$\:{Y}^{t\:-\:n}$, $\:{Y}^{t-\left(n-1\right).q}$, …, $\:{Y}^{t\:-\:1}$], these matrices form a tensor of dimensions *I×J×n*, reshaped into an *I×J* matrix for network input.

Each row of this matrix corresponds to an *n*-day sequence of crime occurrences within one of the *I×J* grid cells of the city. The total number of rows equals the number of grid cells. The input is processed through the LSTM layer, which captures temporal dependencies, and then passed into a fully connected layer with *I×J* columns. Finally, the output is reshaped into an *I×J* matrix, representing the extracted temporal features. This matrix is prepared for integration with the output of the spatio-temporal feature extraction model, facilitating a comprehensive analysis that incorporates both temporal and spatial dimensions of the crime data.

#### Fully connected layer

To assess the impact of external features (such as weather conditions, holidays, weekends, and weekdays) on theft patterns, we employed a fully connected layer. After one-hot encoding, these features were incorporated as model inputs and transformed into a combined feature space for subsequent layers. This approach allows us to directly investigate the influence of external features on predicting theft patterns.

After fusing these three modules, the model’s performance is evaluated on the validation dataset. This allows for hyperparameter tuning to optimize performance. Subsequently, the final performance is assessed using the test set, comparing results across different grid cell dimensions.

### Evaluation metrics

We evaluated the model using RMSE, mean hit rate, and Predictive Accuracy Index (PAI)^49^. RMSE measures the error between predicted and actual values. The mean hit rate shows the proportion of correctly identified hotspot areas, and PAI indicates how well events are clustered spatially. We set coverage thresholds for hotspot areas at 5%, 10%, and 20%. The formulas for these metrics are provided below:

3 $$RMSE=\sqrt {\frac{1}{n}\mathop \sum \limits_{{i=1}}^{n} {{\left( {{{\hat {y}}_i} - {y_i}} \right)}^2}}$$

4 $$Mean~hit~rate~=~\frac{1}{n}~\mathop \sum \limits_{{i=0}}^{n} \left( {\frac{{n_{t}^{*}}}{{N_{t}^{*}}}} \right)*100~$$

5 $$PAI=\left( {\frac{{{n^*}}}{{{N^*}}}/\frac{a}{A}} \right)~~$$

In these equations, *n* is the total number of days across all grid cells, ${\hat {y}_i}$ denotes the predicted value, and ${y_i}~$ represents the ground truth. ${n^*}$ is the number of predicted crimes within coverage areas, while ${N^*}$ is the total number of actual crimes in the study area. Variable *a* represents the predicted hotspots area, and *A* represents the total area of the study region.

#### Model training and evaluation

Learnable parameters are initialized with the default settings in Keras^50^. For the ST-ResNet model, Convolution1 (Conv1) and all subsequent units utilize 64 3 × 3 filters, while Conv2 employs a single 3 × 3 filter, as depicted in Table 3. Additionally, the temporal module consists of a single-layer LSTM and two fully connected layers for training on external features.

Other hyperparameters are selected through a grid search method to optimize our model. The number of Res-units is varied among 2, 4, and 6. For training the LSTM, lag intervals of 7, 14, and 30 days are tested, and the number of LSTM units is chosen from 64, 256, and 512. The number of neurons in the dense layer is also selected accordingly. The Adam optimizer is employed for optimization, with batch sizes of 8, 16, and 32.

Approximately 90% of the data is used for training and evaluating our network, while the remaining 10% is reserved for testing purposes. Subsequently, the model is trained on the entire training dataset for a fixed number of epochs (e.g., 100, 200 epochs), with an early stopping strategy implemented to prevent overfitting.

**Table 3:** Details of the convolutional neural network and the remaining units in 500 m grid size.

Layer name	Output size	Near	Period	Trend
Convolutional 1	70*84	64 (3*3)	64 (3*3)	64 (3*3)
Resnet (2:6)	70*84	$$\left[ {\begin{array}{*{20}{c}} {64\left( {3{\text{*}}3} \right)} \\ {64\left( {3{\text{*}}3} \right)} \end{array}} \right]$$	$$\left[ {\begin{array}{*{20}{c}} {64\left( {3*3} \right)} \\ {64\left( {3*3} \right)} \end{array}} \right]$$	$$\left[ {\begin{array}{*{20}{c}} {64\left( {3*3} \right)} \\ {64\left( {3*3} \right)} \end{array}} \right]$$
Convolutional 2	70*84	1 (3*3)	1 (3*3)	1 (3*3)

### Experiment setup

Initially, the model’s overall accuracy was assessed using the RMSE metric, which quantifies the model’s performance across the entire study area. To enhance the reliability of the RMSE results, each model was run five times, and the average RMSE values were computed. Additionally, the model’s ability to identify crime hotspots was evaluated using Hit Rates and the PAI. Hit Rates indicate the proportion of correctly identified hotspots, while PAI provides a more precise assessment of the accuracy in predicting hotspots.

We tested four progressively enhanced configurations of the ST-ResNet network, each incorporating various features and improvements. Furthermore, we evaluated a comprehensive model that integrates all these enhancements. These configurations were applied across three grid sizes (2000 m, 1000 m, and 500 m) to assess their impact on prediction accuracy. The baseline model, ST-RN, served as our reference point. Here, ST-RN(P) enhances this baseline model by incorporating the daily Euclidean distance of each crime to the nearest park as an environmental feature. ST-RN(E) adds external factors to the baseline model. ST-RN(L) integrates LSTM with ST-ResNet to better capture temporal effects. Finally, the proposed framework combines all these features and improvements into a comprehensive model that leverages the strengths of each enhancement.

To further validate the impact of each enhancement, we conducted structured ablation experiment by systematically removing one feature or module at a time from the proposed model. Specifically, we tested the impact of excluding the park proximity feature (W/O(P)), omitting the auxiliary external variables such as weather and holidays (W/O(E)), and removing the LSTM-based temporal module (W/O(L)). These ablated versions were evaluated alongside the baseline ST-RN model and its progressively improved variants to isolate the contribution of each module.

## Results

In this section, we present model performance using both additive (from base ST-RN upward) and subtractive (from the proposed model downward) ablation strategies to isolate the contribution of each component across different spatial resolutions (Table 4). We then compare the performance and the efficiency of the proposed model with several baseline models (see Supplementary Table A1 and A2).

As shown in Table 4, at the 2000-meter resolution, the proposed model achieved the best performance with an RMSE of 0.9785, representing a 14.15% improvement over the baseline ST-RN (1.1399). Among the ablated variants, removing the LSTM module resulted in the largest performance drop (RMSE = 1.1029), highlighting its critical role in capturing long-term temporal patterns at coarser spatial scales. The removal of external variables (W/O(E): 1.0596) and the park proximity feature (W/O(P): 1.077) also led to lower accuracy. Importantly, adding the LSTM module to the baseline ST-RN architecture also led to substantial gains (ST-RN(L): 0.9863), demonstrating the positive impact of incorporating temporal dynamics even without other enhancements. This double evidence—from both removal and addition—strongly confirms that temporal modeling is the most influential component at this resolution. In terms of hotspot identification, the proposed model reached hit rates of 40.7%, 59.5%, and 82.3% at the 5%, 10%, and 20% thresholds, respectively. It also improved the PAI by 0.13%, reflecting better precision in hotspot detection while maintaining spatial efficiency.

At the 1000-meter resolution, the model continued to outperform other configurations, achieving an RMSE of 0.4762, a 4.76% improvement over the baseline (0.5000). The LSTM-enhanced variant (ST-RN(L): 0.4828) once again showed clear improvements over the baseline, confirming the effectiveness of temporal modeling at medium spatial scales. Similar to the 2000-meter grid, removal of the LSTM from the proposed model caused the most significant accuracy drop, further reinforcing its importance. Conversely, adding LSTM to the baseline network also led to notable improvements, indicating that even at moderate resolutions, capturing sequential crime trends substantially enhances prediction accuracy. Additionally, ST-RN(P), which incorporates the park proximity feature, performed competitively in hotspot identification—especially at the 5% and 10% levels—highlighting the complementary value of environmental context at this scale where both spatial detail and temporal variation are relevant.

Further reducing the grid size to 500 m resulted in achieving the lowest RMSE of 0.2215, marking a 6.34% improvement. This result suggests that the proposed model performs better with finer grid sizes and higher spatial resolution. At the 5% and 10% levels, the proposed model achieved Hit Rates of 62.3193 and 79.9403, respectively, outperforming the ST-RN(P) model. However, at the 20% level, the ST-RN(P) model excelled with a score of 89.0886. These findings indicate that while the proposed model is more effective at identifying higher-density areas, the ST-RN(P) model is better at detecting lower-density crime spots. Additionally, the ST-RN(P) model’s 0.44% improvement in the PAI index underscores the significant role that environmental features play in enhancing model accuracy. While the overall model remained dominant, the performance differences among variants became narrower, possibly due to the increased spatial granularity capturing localized patterns more effectively. Removing the park proximity feature (W/O(P)) notably reduced hotspot prediction accuracy at the 20% threshold, underscoring the importance of dynamic environmental features—especially public spaces—in fine-scale crime prediction.

**Table 4:** Performance comparison of different ST-ResNet configurations.

Grid	Metric	ST-RN	ST_RN(*P*)	ST_RN(E)	ST_RN(L)	Proposed model	W/O(*P*)	W/O(E)	W/O(L)
2000									
	RMSE	1.1399 ± 0.093	1.1180 ± 0.029	1.0757 ± 0.043	0.9863 ± 0.022	0.9785 ± 0.005	1.077 ± 0.05	1.0596 ± 0.065	1.1029 ± 0.028
	5%	40.4596	40.8050	40.2719	40.5319	40.7293	40.6370	40.3869	40.3734
	10%	59.2649	59.5459	59.0474	59.6239	59.5483	59.4945	59.4342	59.3790
	20%	82.1596	82.2398	82.2396	82.1752	82.2714	82.5303	82.1726	82.1510
	PAI	4.1408	4.1448	4.1448	4.1416	4.1464	4.1595	4.1415	4.1404
1000									
	RMSE	0.5000 ± 0.024	0.4995 ± 0.005	0.4909 ± 0.014	0.4828 ± 0.005	0.4762 ± 0.006	0.4942 ± 0.005	0.4944 ± 0.003	0.4996 ± 0.007
	5%	47.2329	49.1717	48.7620	48.6057	47.6079	48.8072	48.7218	48.6112
	10%	66.7444	68.1849	67.7449	67.7788	67.2207	67.8417	67.7915	67.6524
	20%	86.8073	87.2280	86.8698	86.8912	87.2299	87.0831	86.8960	87.1926
	PAI	4.3403	4.3614	4.3434	4.3445	4.3614	4.3541	4.3448	4.3596
500									
	RMSE	0.2365 ± 0.004	0.2284 ± 0.002	0.2305 ± 0.001	0.2324 ± 0.002	0.2215 ± 0.0005	0.2308 ± 0.001	0.2281 ± 0.002	0.2247 ± 0.001
	5%	61.5344	62.2208	61.7956	61.8552	62.3193	61.5404	62.2082	62.2547
	10%	79.3976	80.0369	79.6653	79.5303	79.9403	79.2326	79.8934	80.1062
	20%	88.6974	89.0886	88.8842	88.4136	88.7402	88.1339	88.6536	89.1570
	PAI	4.4348	4.4544	4.4442	4.4206	4.4370	4.4066	4.4326	4.4578

**Fig. 4:**
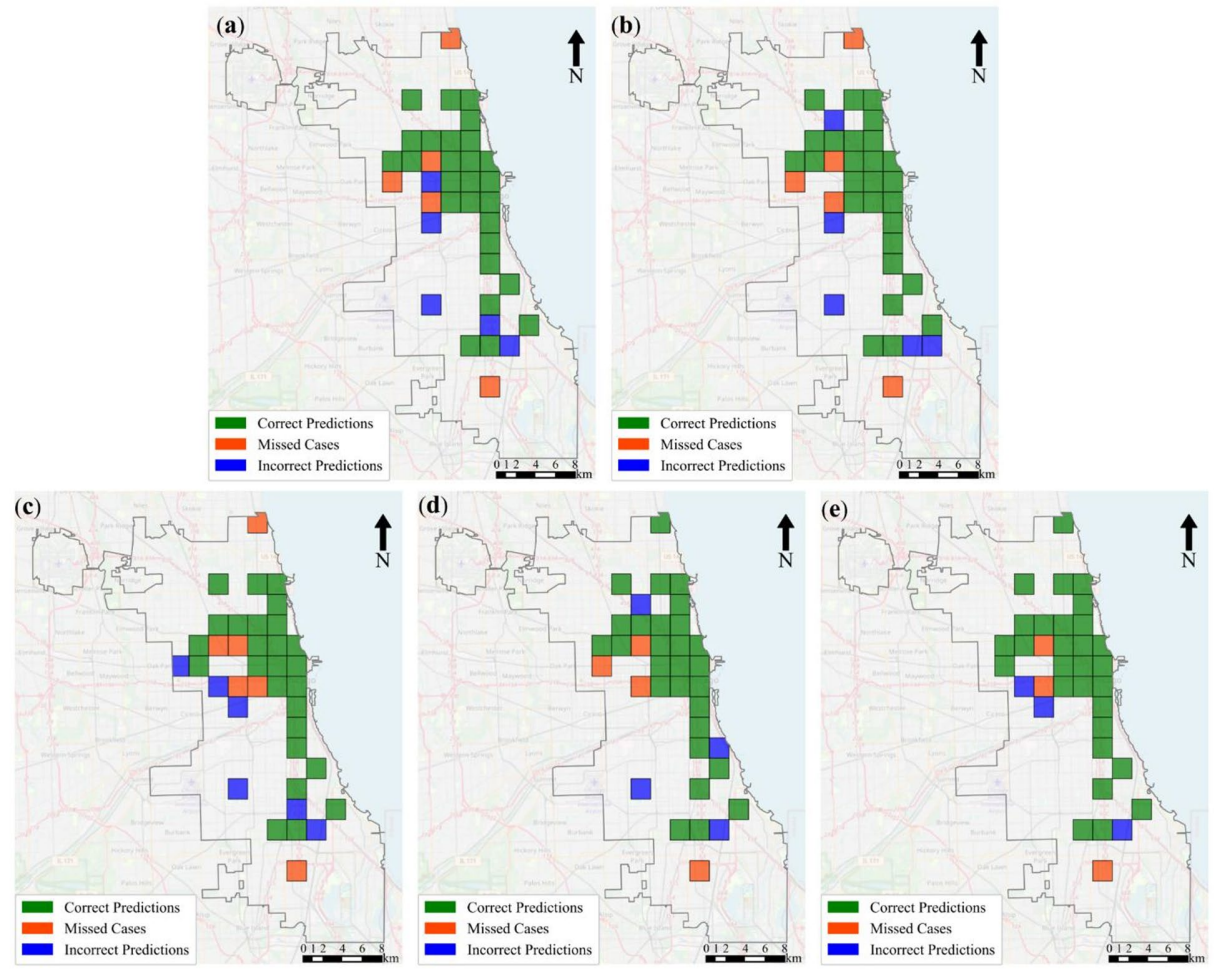
Model hotspot prediction in 20% coverage area on day 60th of test data. **(a)** ST-RN **(b)** ST-RN(L), **(c)** ST-RN(E), **(d)** ST-RN(P), **(e)** Proposed framework.

While Hit Rate is a simple and general numerical metric, we utilized a more detailed analysis and visualization tool to evaluate the model’s performance in predicting hotspot areas. As shown in Fig. 4, this tool breaks down the percentage of correct predictions (green cells), missed cases (orange cells), and incorrect predictions (blue cells), offering a comprehensive assessment of the model’s ability to accurately identify crime hotspots. For visualizing the predicted locations, we used Google Street Maps. The 60th day of the test data was selected to assess the model’s performance with 20% coverage in predicting hotspots. The baseline ST-RN model (Fig. 4a) and its variant ST-RN(L) (Fig. 4b) showed comparable performance. In contrast, ST-RN(E) (Fig. 4c) performed worse than the baseline. The ST-RN(P) model (Fig. 4d) achieved a clear improvement over ST-RN(E) and also outperformed the baseline, while the proposed framework (Fig. 4e) yielded the best results, with the highest number of correct predictions and the fewest errors. The proposed model, compared to the baseline ST-RN, accurately predicted two cells, each covering 2 square kilometers, and produced fewer incorrect predictions. Additionally, the proposed model had fewer false predictions on this day. This visual comparison confirms the effectiveness of integrating both temporal dynamics and environmental features in improving predictive accuracy.

**Fig. 5:**
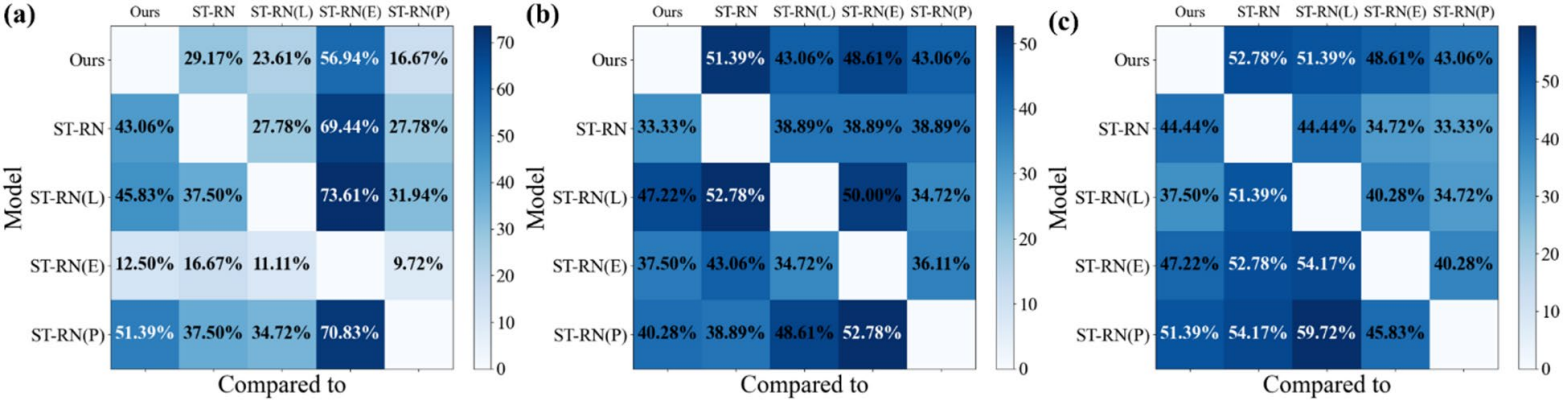
The percentage of correctly predicting the location of hotspots in 20% coverage level across different enhancements: (**a**) 2000 m, (**b**) 1000 m, and (**c**) 500 m.

Figure 5 complements this analysis by summarizing the models’ comparative performance over all 72 test days across three spatial resolutions: 2000 m (Fig. 5a), 1000 m (Fig. 5b), and 500 m (Fig. 5c). Each matrix indicates how often each model outperformed the others in identifying correct hotspot locations within the top 20% coverage area.

For example, at a 500-meter resolution, the proposed model outperformed the ST-RN on 38 out of 72 test days, achieving a success rate of 52.78%. In contrast, the ST-RN model performed better on 32 days, recording a success rate of 44.44%. On the remaining two days, both models performed similarly. A similar pattern is observed at 1000 m, where the proposed model again leads with 51.39% success over ST-RN’s 40.28%. Importantly, the 500-meter resolution not only demonstrated the highest overall success rate but also enabled more precise localization of hotspot cells, capturing finer-grained spatial patterns that were less distinguishable in the larger grid sizes. This indicates that finer spatial resolutions, when combined with the proposed model’s architecture, can lead to significantly better identification of exact hotspot locations. This analysis suggests that combining environmental features and temporal networks can significantly improve the accuracy of hotspot location predictions, particularly at finer spatial resolutions.

After evaluating the overall performance of the proposed model, we analyzed the impact of each feature separately to better understand its role in accuracy and efficiency, helping us to better identify the model’s strengths and weaknesses.

**Fig. 6:**
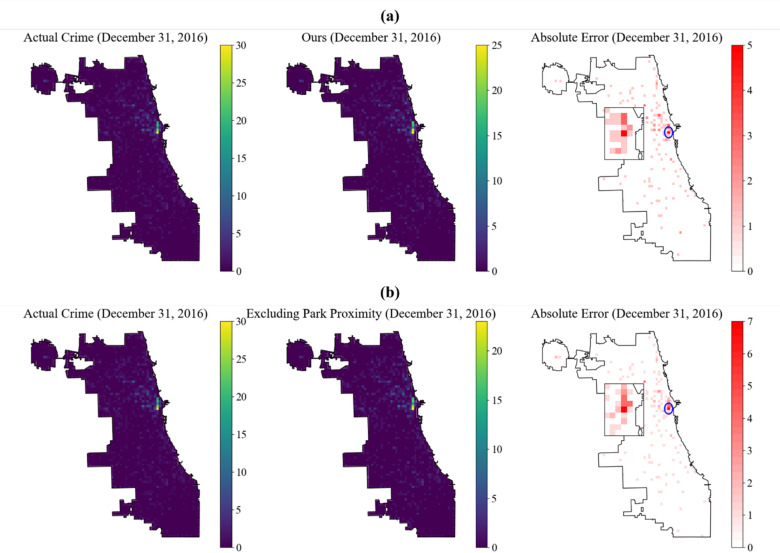
Comparison of predicting crime hotspots: (**a**) with and (**b**) without park proximity.

Including the daily Euclidean distance from the crime location to the nearest park as an environmental feature significantly improved the accuracy of hotspot predictions (Fig. 6a). Statistical analysis showed that removing this feature from the model noticeably increased the Absolute Error (AE), particularly in high-density crime areas, where AE increased from 156 to 162, representing a 6.41% rise (Fig. 6b). Additionally, the model’s RMSE increased to 0.2247 after removing this feature.

Incorporating a temporal network (LSTM) into the model significantly improved prediction accuracy, particularly on weekdays and at higher spatial resolutions (see Supplementary Fig. A1 online). The LSTM network enables the model to better recognize temporal sequences and recurring patterns, which is especially useful in predicting crimes on weekdays where more structured and predictable patterns exist (see Supplementary Fig. A2 online). This feature helped the model accurately detect crime patterns during busier days of the week due to their more structured and predictable nature.

**Fig. 7:**
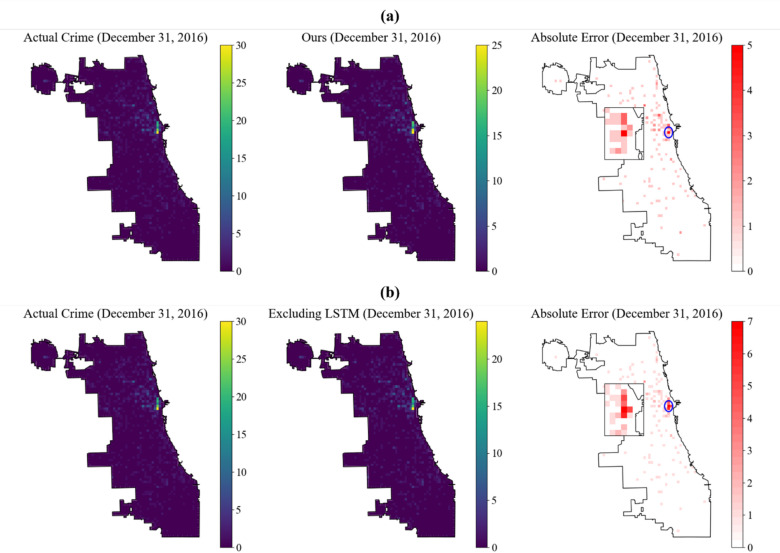
Comparison of predicting crime hotspots: (**a**) with and (**b**) without LSTM network.

To further examine the impact of LSTM, we analyzed the last day of the test data. The results with the LSTM (Fig. 7a) show lower AE and better overall accuracy. When the LSTM was removed (Fig. 7b), the AE increased by 3.85%, particularly in high-density crime areas. This indicates the importance of considering temporal dynamics and utilizing temporal networks in improving model predictions.

**Fig. 8:**
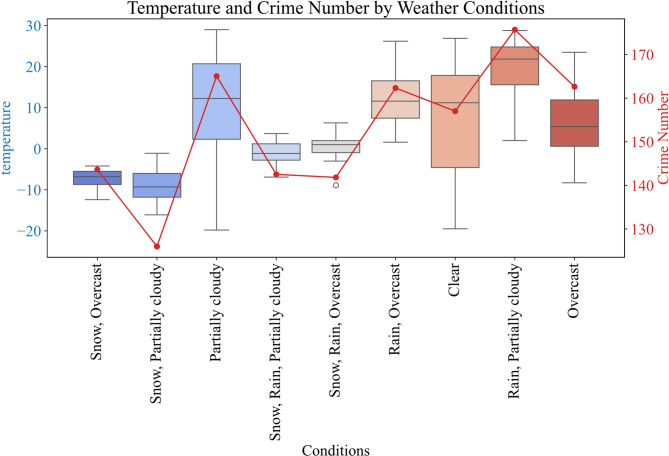
Relationship between weather conditions, temperature, and crime counts.

The analysis of weather conditions revealed that the model performed best on sunny days, while its accuracy slightly decreased on rainy or snowy days. Statistical analysis also showed that temperature significantly correlated with crime numbers (Fig. 8). Higher temperatures were associated with increased crimes, likely due to more outdoor activities and social interactions.

The detailed analysis of each feature showed that while each independently improved model accuracy, combining these features in the comprehensive model led to substantial enhancements in overall performance. Notably, features accounting for environmental and temporal interactions significantly improved the model’s accuracy in predicting crime hotspots, highlighting the importance of contextual factors in crime pattern analysis.

The results also showed that the proposed comprehensive model, which integrates all these features, performs exceptionally well at finer spatial resolutions and under specific conditions, such as weekdays and stable weather. These analyses demonstrate that a multifaceted approach that considers both environmental and temporal features can be a powerful tool for more accurate and effective crime hotspot prediction, reinforcing the value of holistic data integration in predictive modeling.

## Discussions

This study aims to improve crime prevention strategies in Chicago by developing a model for short-term, daily crime prediction, specifically targeting theft. The research addresses gaps in previous studies and enhances police resource allocation, as most prior work has focused on long-term predictions^48^, such as monthly or yearly forecasts^51,52^. By analyzing daily theft incidents and applying advanced statistical methods, this study aims to uncover hidden patterns in the data to predict crime occurrences across different areas of the city. The focus on theft crimes is driven by their relative frequency and the need for accurate, timely predictions to optimize police resource allocation and enhance urban safety. Furthermore, unlike previous research, we benchmarked the proposed model against both traditional baselines and recent SOTA approaches to ensure a fair and comprehensive evaluation.

Previous research has focused on spatial segmentation of cities and the placement of crime counts in grid cells at varying spatial and temporal levels^53^. Additionally, various techniques have been employed to address data sparsity^54,55^. In this study, we adopted the DPIM approach, similar to Wang et al.^11^, to tackle data sparsity and applied it consistently across all baselines and spatial resolutions (coarse, moderate, and fine) to ensure fair comparison. Our findings reveal that most baseline models—including CNN-LSTM, Conv-LSTM, GRU, LSTM, ST-ResNet, Vision Transformer (ViT), and Enhanced ST-GCN— showed their best hotspot prediction accuracy or overall stronger performance at the intermediate 1000 m resolution compared to the finer 500 m scale. This trend is consistent with previous studies, such as Zhang et al.^12^ and Jing et al.^13^, which reported that moderate spatial resolutions often yield more stable outcomes in crime prediction. These results highlight a key limitation in existing models, as those that rely primarily on historical crime counts—without incorporating dynamic environmental features or reinforcement mechanisms—struggle to capture the complex spatio-temporal interactions required for accurate prediction at finer resolutions.

In contrast, the proposed model overcomes these limitations by integrating historical crime data with dynamic environmental features —including dynamic spatio-temporal variables such as daily park proximity and dynamic temporal variables such as meteorological conditions—alongside static contextual variables such as weekday/weekend indicators and holidays. An LSTM module was added to capture sequential dependencies, and an attention mechanism was embedded within the residual units. This integrated design enabled the model to maintain and even improve predictive accuracy at the fine 500 m resolution, where baseline models showed substantial performance drops. Compared to classical architectures, the proposed model achieved significantly higher accuracy, and when benchmarked against recent SOTA approaches such as ViT and Enhanced ST-GCN, it delivered competitive and, in some cases, superior results. Moreover, the 14.15% improvement in RMSE at coarser resolutions shows that incorporating environmental and temporal features not only strengthens performance at the neighborhood level but also enhances robustness across broader urban areas. These findings are consistent with prior research, which suggests that finer spatial resolutions are more effective for identifying the relationship between crime levels and influencing factors in smaller areas^56,57^.

The ablation study further clarified which components contributed most to the model’s performance. The enhanced ST-ResNet (ST-RN), which incorporates an attention mechanism within the residual units across all configurations, achieved the greatest improvements when combined with dynamic environmental features. In particular, adding the daily Euclidean distance of crimes to the nearest park as a key dynamic spatio-temporal feature enabled the ST-RN(P) variant to achieve the highest hotspot prediction accuracy across all baselines and SOTA models, reaching 89.0886% at the 500 m scale. Conversely, removing this feature (W/O(P)) resulted in the largest accuracy drop, confirming its critical role. Notably, these findings align with theories on the impact of public spaces on crime rates^58,59^, as parks and gathering areas can act as focal points where higher population density increases opportunities for crime. By modeling proximity to parks, the proposed framework was better able to identify and predict high-risk areas, underscoring the strong correlation between crime occurrence and public open spaces.

Similarly, according to our findings, adding an LSTM module (ST-RN(L)) significantly improved the model’s ability to capture temporal continuity and behavioral cycles, particularly on weekdays when patterns are more regular. In contrast, baseline spatial networks such as ST-ResNet process temporal data in separate branches before merging them into a three-dimensional tensor at the final stage, a design choice that results in the loss of sequential dependencies. These limitations became evident in the ablation experiment, where removing the LSTM (W/O(L)) caused a substantial drop in performance—especially at coarser resolutions (1000 m and 2000 m)—underscoring its essential role in modeling temporal dynamics. Overall, these results demonstrate that combining dynamic environmental features with sequential modeling through LSTM is fundamental for achieving reliable fine-grained crime prediction.

Despite these strengths, our results also revealed persistent challenges in predicting crime during weekends and holidays. Errors were consistently higher in these periods, aligning with earlier findings^14^. This limitation is likely linked to irregular lifestyle changes on weekends and the influx of tourists during holiday periods, both of which introduce variability that is harder to capture with existing temporal or environmental features. While the integration of LSTM and dynamic context improved accuracy under regular daily cycles, future models will need to account for the non-routine dynamics of weekends and special events to further reduce prediction errors.

Overall, the findings of this study demonstrate that the proposed model can improve hotspot prediction across spatial scales and uncover more complex crime patterns, offering clear value for real-world policing. By enabling law enforcement agencies to allocate resources more effectively, design more targeted prevention strategies, and ultimately enhance public safety, the model addresses both scientific and practical needs. Despite the added training cost, our framework required less time and achieved greater efficiency than hybrid architectures such as Conv-LSTM and CNN-LSTM. Moreover, compared to recent SOTA approaches—including the ViT and Enhanced ST-GCN—the proposed model not only delivered competitive and sometimes superior predictive accuracy but also required shorter training times, highlighting its advantage in both performance and efficiency. This balance underscores the practical importance of model selection and the need to align predictive accuracy with available resources in real-world deployment contexts.

### Limitations

Despite the strong potential of the proposed model for spatio-temporal crime prediction, several limitations remain that need to be addressed. Although the model has demonstrated an overall improvement in accuracy, challenges persist in refining its precision. Specifically, the modest increase in the PAI index and the relatively higher AE in crime hot spots indicate difficulties in further enhancing accuracy. These challenges may be attributed to significant fluctuations in crime rates in North Chicago, especially near major tourist attractions like Lake Michigan, which are heavily influenced by local conditions.

The social dynamics in these areas are complex due to the daily influx of tourists, leading to substantial variations in theft crime rates. Incorporating more social factors into the model is therefore essential to better capture these diverse crime patterns.

Additionally, these limitations may stem from the inherent complexities of crime data or the need for more advanced algorithms to improve pattern recognition. The model’s reduced accuracy during adverse weather conditions also suggests that environmental factors like temperature and humidity need to be more accurately incorporated to enhance prediction performance.

Furthermore, while Transformer-based models have gained popularity in various domains, their suitability for spatio-temporal crime prediction remains debatable. Prior research has highlighted critical limitations of Transformers in handling hierarchical reasoning and compositional tasks^60,61^, both of which are fundamental in crime pattern modeling. In our experiments, a spatio-temporal Transformer was also attempted; however, its quadratic computational complexity made it infeasible at high spatial resolutions, and its predictive performance was unsatisfactory. Given these constraints, we focused on CNN- and LSTM-based architectures, which provide a better balance between accuracy and computational efficiency. Looking ahead, future research may explore hybrid frameworks that leverage attention mechanisms while addressing these limitations.

## Conclusion

This study presents an enhanced spatio-temporal prediction framework that addresses key limitations of existing models. Unlike previous approaches that showed peak accuracy at the intermediate 1000 m resolution, the proposed framework achieves its strongest performance at the finer 500 m scale, which is most relevant for operational policing.

The novelty of our work lies in three main contributions. First, we introduce daily park proximity as a dynamic spatio-temporal environmental feature, marking its first use in crime prediction and capturing the influence of urban public spaces on crime patterns. Second, we embed an attention mechanism within residual units, enabling the model to effectively handle sparsity and irregularity in crime data. Third, we integrate these innovations with LSTM-based sequential modeling and a standardized DPIM preprocessing pipeline, ensuring robustness and fairness across different spatial resolutions.

Through this integrated design, the proposed framework consistently outperforms classical baselines (e.g., CNN-LSTM, Conv-LSTM, GRU, and ST-ResNet) and delivers competitive, and in some cases superior, results compared with recent SOTA methods such as ViT and Enhanced ST-GCN. Importantly, it achieves these gains with shorter training time than hybrid and SOTA architectures, highlighting both methodological advancement and computational efficiency.

Overall, this research provides a scalable and practical tool for crime prediction, advancing the methodological frontier while offering direct value for law enforcement through more precise hotspot identification, optimized patrol planning, and effective resource allocation at neighborhood scales.

## Supplementary Information

Below is the link to the electronic supplementary material.

Supplementary Material 1

## Data Availability

The datasets used and analyzed during the current study are available from the corresponding author upon reasonable request.
