# Supplementary material for: Hybrid ST-ResNet and LSTM approach for precise crime hotspot prediction
Source: Sci Rep. 2025 Nov 19;15:40754. doi: 10.1038/s41598-025-24559-7 (PMC12630685; doi:10.1038/s41598-025-24559-7)
Supplement: Supplementary file 1 — Supplementary Material 1 [file 41598_2025_24559_MOESM1_ESM.docx]

**Hybrid ST-ResNet and LSTM Approach for Precise Crime Hotspot Prediction**

Nasim Shahmoradi^1^, Ali Asghar Alesheikh^1,2, *^, Ali Jafari^1^, Aynaz Lotfata^3^

^1^Department of Geospatial Information Systems, Faculty of Geodesy and Geomatics Engineering, K. N. Toosi University of Technology, Tehran, Iran- Emails: n.shahmoradi@email.kntu.ac.ir; [alesheikh@kntu.ac.ir](mailto:alesheikh@kntu.ac.ir)*;* [a.jafari2@email.kntu.ac.ir](mailto:a.jafari2@email.kntu.ac.ir)

^2^Geospatial Big Data Computations and Internet of Things (IoT) Lab, K. N. Toosi University of Technology, Tehran, Iran; alesheikh@kntu.ac.ir

^3^Department of Pathology, Microbiology, and Immunology, School of Veterinary Medicine, University of California, Davis, USA. Email: [alotfata@gmail.com](mailto:alotfata@gmail.com%20)

*Corresponding author: Ali Asghar Alesheikh, [alesheikh@kntu.ac.ir](mailto:alesheikh@kntu.ac.ir)

**Supplementary Information**

**Supplementary File A**

**Table A1** includes the baseline methods:

- **GRU**: This model employs a Gated Recurrent Unit (GRU) layer to capture temporal patterns in the crime data. GRU is designed to efficiently model sequential dependencies by using gating mechanisms that control the flow of information through time steps. The model was trained using a single GRU layer across all three spatial resolutions (2000m, 1000m, and 500m).
- **LSTM**: This model was trained on data from all three spatial resolutions (2000m, 1000m, and 500m) using one LSTM layer to capture temporal dependencies in the crime data.
- **CNN-LSTM**: This hybrid model combines Convolutional Neural Networks (CNN) for spatial feature extraction with LSTM for temporal sequence modeling. Initially, CNN layers extract spatial patterns from the input grids, and the resulting features are passed to an LSTM layer to learn temporal dynamics. The model was trained at all three spatial resolutions to capture complex spatio-temporal relationships in crime data.
- **Convolutional LSTM (Conv-LSTM)**: This variant of the LSTM model replaces the fully connected layer with a convolutional layer, enhancing the model’s ability to capture spatio-temporal features. We employed two Conv-LSTM layers.
- **ST-ResNet**: This model follows the ST-ResNet proposed by Zhang et al. (2017), which captures short-term, periodic, and long-term spatial dependencies through multiple residual units. Each residual unit applies convolutional layers to learn spatial features from crime grids, while the residual connections help stabilize deep learning. In line with the original design, external features such as weather conditions and calendar indicators (weekdays, weekends, holidays) were incorporated via a fully connected layer.
- **Improved Vision Transformer (ViT)**: The vision transformer model applies a patch-based tokenization of the crime grid, where each grid patch is treated as a token and processed by transformer encoder layers. The self-attention mechanism enables the model to capture long-range spatial dependencies. The model was trained across resolutions of 2000m, 1000m, and 500m to evaluate its effectiveness in spatio-temporal hotspot detection.
- **Enhanced ST-GCN**: This graph-based model builds a spatio-temporal graph where each grid cell is treated as a node connected to its neighbors. Weighted adjacency matrices (including one-hop and two-hop supports) allow the model to capture both local and higher-order spatial dependencies. Temporal convolutional blocks with dilation are applied to extract long-term temporal dynamics. Residual connections, layer normalization, and dropout improve stability. This enhanced ST-GCN was trained and evaluated at all three resolutions.

The proposed model was benchmarked against a broad set of baselines, including recurrent models (GRU, LSTM), hybrid convolutional–recurrent models (CNN-LSTM, Conv-LSTM), the widely used ST-ResNet, and more recent state-of-the-art approaches such as the Vision Transformer (ViT) and an Enhanced ST-GCN. To ensure fairness, all models were trained under the same data preparation pipeline, with DPIM applied to address sparsity. This comparison (**Table A1**) spans both classical and state-of-the-art architectures and was conducted across three spatial resolutions (2000 m, 1000 m, and 500 m) to evaluate robustness and adaptability at different levels of spatial granularity.

At the 2000 m resolution, Enhanced ST-GCN recorded the lowest errors (MAE = 0.0771, RMSE = 0.2473), while Conv-LSTM achieved the highest R² (0.93) and ST-ResNet and CNN-LSTM also showed strong explanatory power (R² ≈ 0.90). ViT lagged behind with a lower R² (0.74). Although the proposed model did not minimize error at this coarse scale (MAE = 0.3796, RMSE = 0.9785), it achieved the best hotspot-targeting metrics (hit-rates at 5%, 10%, 20% and PAI), reflecting its advantage in spatial prioritization even under limited coverage.

At the 1000 m resolution, most baselines reached their peak hotspot prediction performance. Enhanced ST-GCN provided the lowest error values (MAE = 0.0763, RMSE = 0.3282), ST-ResNet led at the 20% hit-rate and PAI, and Conv-LSTM maintained strong accuracy (R² = 0.86). The proposed model attained the highest overall explanatory power (R² = 0.93) and remained competitive across hotspot metrics, indicating robustness at this intermediate scale.

At the 500 m resolution, the proposed model clearly outperformed all baselines, achieving the lowest error (MAE = 0.0327, RMSE = 0.2215) and the highest R² (0.93). In contrast, Enhanced ST-GCN showed increased error (RMSE = 0.3736, R² = 0.79) and ViT declined further (RMSE = 0.5676, R² = 0.54). The proposed model also delivered the top hotspot localization at 5% and 20% coverage and the highest PAI (4.4370). These findings confirm that while most baselines peak at 1000 m, the proposed model uniquely improves further at the finer 500 m resolution, sustaining high accuracy where neighborhood-level hotspot prediction is most critical.

Together, these findings illustrate the capability of the proposed model in both accuracy and spatial targeting, making it a robust and reliable approach for short-term urban crime prediction tasks.

**Table A2** presents a comparative analysis of model efficiency between the proposed method and six baseline models (GRU, LSTM, CNN-LSTM, Conv-LSTM, ViT, and ST-GCN) across three spatial resolutions (500 m, 1000 m, and 2000 m). Two key performance indicators are reported: average training time per epoch (in seconds) and average inference time per sample (in milliseconds). All experiments were conducted using an NVIDIA RTX 3060 GPU, except for Conv-LSTM at 500 m resolution, which exceeded GPU memory limitations and was therefore run on a high-memory CPU (140 GB RAM).

The results show that Conv-LSTM exhibits the highest computational cost at all resolutions, requiring substantially longer training and inference times. Enhanced ST-GCN and ViT also demonstrate high computational overhead, especially at finer spatial resolutions. In contrast, the proposed model maintains a favorable balance between efficiency and accuracy, with training times significantly lower than Conv-LSTM, CNN-LSTM, ViT, and ST-GCN.

Notably, at the 500 m resolution—where prediction is most computationally demanding—the proposed model achieved efficient runtimes (4 s/epoch, 1 ms/sample), far outperforming CNN-LSTM (9.76 s/epoch, 6.58 ms/sample), Conv-LSTM (750 s/epoch, 12 ms/sample, CPU), Enhanced ST-GCN (118.78 s/epoch, 58.81 ms/sample), and ViT (11.76 s/epoch, 9.41 ms/sample). This highlights the practicality of the proposed model for real-world applications requiring high-resolution hotspot prediction with manageable computational costs.

**Table A1.** Comparison of the proposed network with baselines

|  |  | **GRU** | **LSTM** | **CNN-LSTM** | **Conv-LSTM** | **St-resnet** | **ViT** | **Enhanced ST-GCN** | **Proposed model** |
| --- | --- | --- | --- | --- | --- | --- | --- | --- | --- |
| **Grid** | **Metric** |  |  |  |  |  |  |  |  |
| **2000** |  |  |  |  |  |  |  |  |  |
|  | MAE | 1.0344 | 1.006 | 0.9683 | 0.4911 | 0.6451 | 0.1427 | 0.0771 | 0.3796 |
|  | RMSE | 2.6220 | 2.6156 | 2.2646 | 1.1171 | 1.3068 | 0.3546 | 0.2473 | 0.9785 |
|  | R^2^ | 0.8400 | 0.8700 | 0.9000 | 0.9300 | 0.9000 | 0.7444 | 0.8973 | 0.9100 |
|  | 5% | 38.9905 | 39.3476 | 39.4249 | 40.4957 | 40.2087 | 38.5511 | 39.9776 | 40.7293 |
|  | 10% | 56.3835 | 56.4860 | 56.6810 | 59.0528 | 58.6886 | 55.8158 | 58.5482 | 59.5483 |
|  | 20% | 78.6728 | 79.9695 | 80.0611 | 81.5606 | 81.3549 | 79.3675 | 81.4331 | 82.2714 |
|  | PAI | 3.9651 | 4.0304 | 4.0350 | 4.1106 | 4.1002 | 4.0001 | 4.1042 | 4.1464 |
| **1000** |  |  |  |  |  |  |  |  |  |
|  | MAE | 0.4328 | 0.4294 | 0.4321 | 0.1768 | 0.3271 | 0.1770 | 0.0763 | 0.1265 |
|  | RMSE | 1.2261 | 1.2101 | 1.1620 | 0.5677 | 0.6513 | 0.4247 | 0.3282 | 0.4762 |
|  | R^2^ | 0.7200 | 0.7300 | 0.7500 | 0.8600 | 0.8100 | 0.6564 | 0.8387 | 0.9300 |
|  | 5% | 41.6057 | 42.2256 | 42.8189 | 48.2081 | 48.1070 | 42.3307 | 48.9414 | 47.6079 |
|  | 10% | 58.7329 | 59.1785 | 60.1282 | 67.1699 | 67.2921 | 59.3538 | 67.4226 | 67.2207 |
|  | 20% | 80.6314 | 80.7663 | 81.9696 | 86.7216 | 87.5674 | 80.8923 | 86.2821 | 87.2299 |
|  | PAI | 4.0315 | 4.0383 | 4.0984 | 4.3360 | 4.3783 | 4.0446 | 4.3141 | 4.3614 |
| **500** |  |  |  |  |  |  |  |  |  |
|  | MAE | 0.1960 | 0.1768 | 0.1829 | 0.0611 | 0.0941 | 0.1966 | 0.0602 | 0.0327 |
|  | RMSE | 0.6716 | 0.6678 | 0.5868 | 0.3054 | 0.2705 | 0.5676 | 0.3736 | 0.2215 |
|  | R^2^ | 0.6500 | 0.4900 | 0.5200 | 0.7800 | 0.7600 | 0.5400 | 0.7947 | 0.9300 |
|  | 5% | 44.4971 | 43.4824 | 44.3384 | 59.9986 | 45.8139 | 41.2895 | 62.2994 | 62.3193 |
|  | 10% | 58.9565 | 58.5707 | 58.8511 | 79.9717 | 61.6927 | 55.3278 | 80.084 | 79.9403 |
|  | 20% | 71.3423 | 68.4473 | 75.0781 | 86.2400 | 80.0157 | 68.3703 | 88.621 | 88.7402 |
|  | PAI | 3.5671 | 3.4223 | 3.7539 | 4.3120 | 4.0007 | 3.4185 | 4.4310 | 4.4370 |

**Table A2.** Efficiency and Parameter Comparison of the Proposed and Baseline Models

| **Grid** | **Model** | **Training Time / Epoch (s)** | **Inference Time / Sample (ms)** | **Hardware used** |
| --- | --- | --- | --- | --- |
| **2000** |  |  |  |  |
|  | **GRU** | 0.48 | 0.01 | GPU (3060) |
|  | **LSTM** | 0.6 | 0.01 | GPU (3060) |
|  | **CNN-LSTM** | 1 | 0.33 | GPU (3060) |
|  | **Conv-LSTM** | 11 | 2.625 | GPU (3060) |
|  | **ViT** | 4.25 | 2.09 | GPU (3060) |
|  | **ST-GCN** | 5.70 | 3.96 | GPU (3060) |
|  | **Proposed model** | 1.92 | 0.16 | GPU (3060) |
| **1000** |  |  |  |  |
|  | **GRU** | 0.54 | 0.01 | GPU (3060) |
|  | **LSTM** | 0.64 | 0.33 | GPU (3060) |
|  | **CNN-LSTM** | 2.86 | 1.95 | GPU (3060) |
|  | **Conv-LSTM** | 46.94 | 5.6 | GPU (3060) |
|  | **ViT** | 11.13 | 8.48 | GPU (3060) |
|  | **ST-GCN** | 16.78 | 11.29 | GPU (3060) |
|  | **Proposed model** | 2.79 | 0.6 | GPU (3060) |
| **500** |  |  |  |  |
|  | **GRU** | 0.76 | 0.01 | GPU (3060) |
|  | **LSTM** | 0.75 | 0.2 | GPU (3060) |
|  | **CNN-LSTM** | 9.76 | 6.58 | GPU (3060) |
|  | **Conv-LSTM** | 750 | 12 | CPU )140GB ( |
|  | **ViT** | 11.76 | 9.41 | GPU (3060) |
|  | **ST-GCN** | 118.78 | 58.81 | GPU (3060) |
|  | **Proposed model** | 4 | 1 | GPU (3060) |

**Figure A1** illustrates the comparison between the actual total crimes and the predicted total crimes for weekdays and weekends. It presents two scenarios: one that includes the LSTM network's predictions and another that excludes it. The trend lines indicate how accurately the proposed model predicts crime occurrences during different days of the week. The solid blue line represents the actual total crimes reported, providing a baseline for comparison. The dashed red line shows the predictions made by the model that incorporates the LSTM network, highlighting its performance in capturing crime trends. The dashed green line reflects the predictions made without the influence of the LSTM model, allowing for an assessment of the LSTM's impact on overall prediction accuracy.

**Figure A2** presents a bar chart comparing the actual total crime counts and the predicted total crime counts for each day of the week, based on the test data. The blue bars represent actual crime counts, while the predicted crime counts are shown in red color.


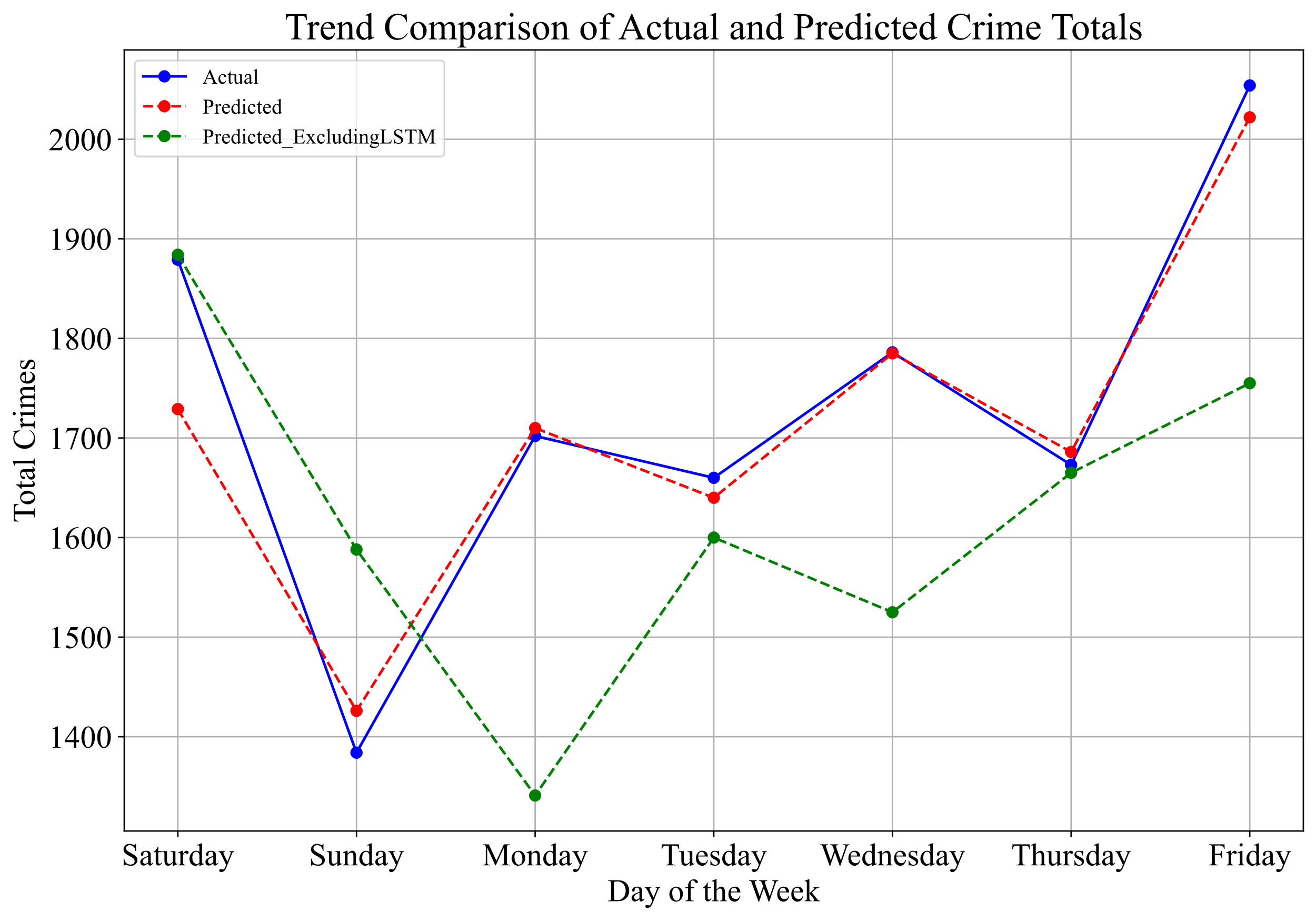


**Fig. A1.** Trend Comparison of Actual and Predicted Total Crimes on Weekdays and Weekends, Including and Excluding LSTM network


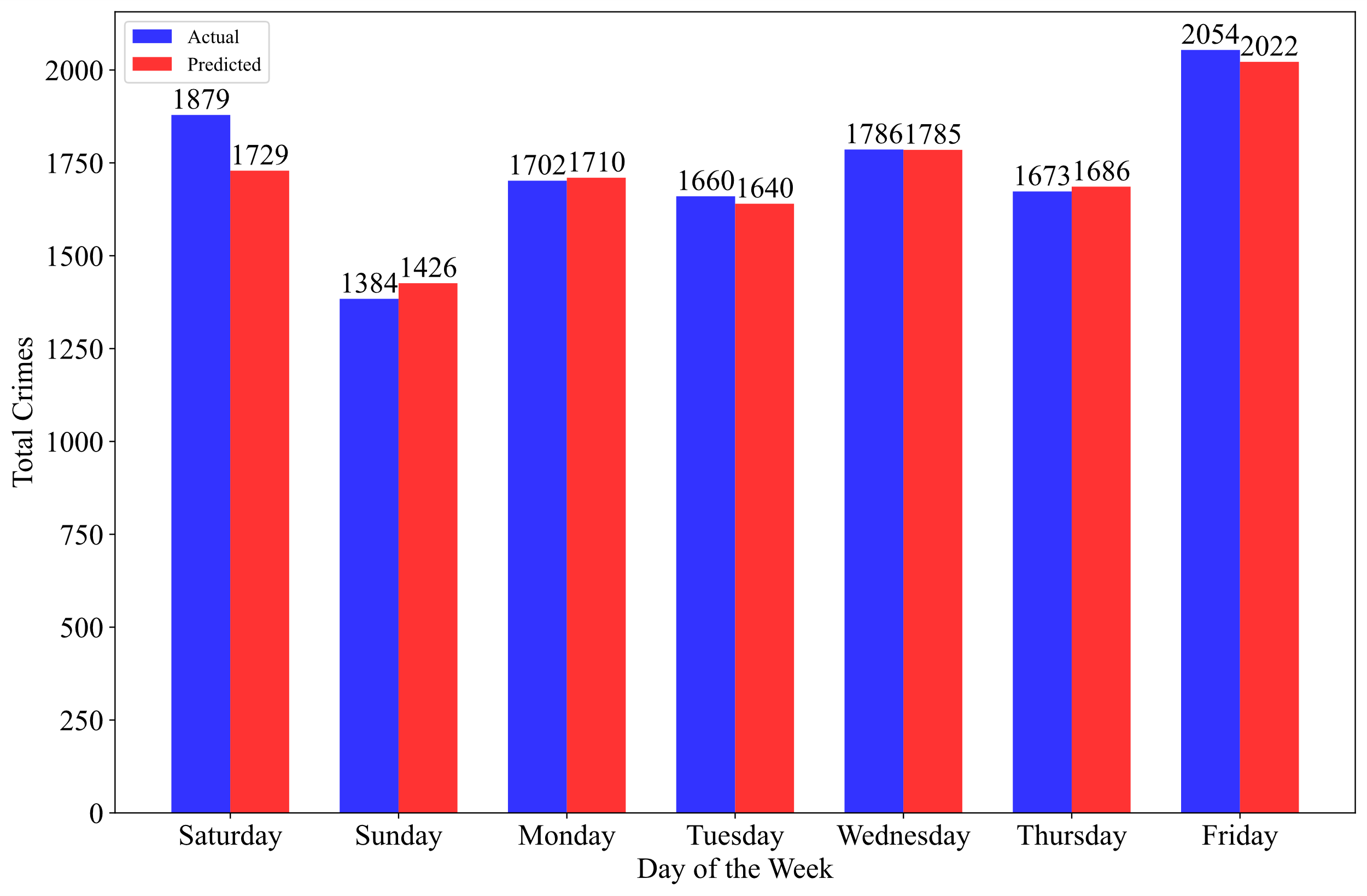


**Fig. A2.** Comparison of actual versus predicted total crime counts by day of the week based on test data
